# Comparison of Knowledge About Induced Pluripotent Stem Cells in Relation to Gender Among Healthcare Professionals and in the General Population

**DOI:** 10.7759/cureus.66821

**Published:** 2024-08-13

**Authors:** Jasna M Gacic, Sanja R Rascanin, Mirjana R Jovanovic, Srdjan S Nikolovski, Nina Jovanovic, Jelena Petkovic, Nebojsa Zdravkovic, Olivera Djokic, Nemanja K Rancic

**Affiliations:** 1 Surgery, Clinical Hospital Center “Bezanijska kosa”, School of Medicine, University of Belgrade, Belgrade, SRB; 2 Faculty of Medical Sciences, University of Kragujevac, Kragujevac, SRB; 3 Psychiatry, Psychiatric Clinic, Clinical Center "Kragujevac", Kragujevac, SRB; 4 Pathology and Laboratory Medicine, Cardiovascular Research Institute, Loyola University Chicago, Maywood, USA; 5 Ophthalmology, Eye Institute “Knezovic“, Zagreb, HRV; 6 Medical Biochemistry, General Hospital “Stefan Visoki”, Smederevska Palanka, SRB; 7 Cardiology, Institute for Cardiovascular Diseases “Dedinje”, Belgrade, SRB; 8 Center for Clinical Pharmacology, Military Medical Academy, Faculty of Medicine of the Military Medical Academy, University of Defence, Belgrade, SRB

**Keywords:** stem cells, induced pluripotent stem cells, knowledge, gender, questionnaire

## Abstract

Introduction: Induced pluripotent stem cells (iPSCs) are derived by reprogramming adult somatic cells using a forced expression of four specific transcription factors in a highly controlled artificial environment. The aim of this paper is to examine the knowledge about these cells of the general population and the population of health workers in relation to gender.

Methods: The research was designed as a cohort study conducted with a validated questionnaire to assess knowledge about iPSCs. Respondents were people over 18 years of age on the territory of the cities of Belgrade and Kragujevac in Serbia.

Results: The study surveyed a total of 1,047 respondents, 560 (53.5%) women and 487 (46.5%) men. Statistically significant differences were observed for both genders. Women from both populations were better informed, more often agreed to treatment with iPSCs, more often supported further research, and were willing to take further education about iPSCs.

Conclusion: Comparing men and women from both populations, we found that men and women health workers showed greater knowledge compared to the general population. Level of knowledge and attitudes of the public can have multiple effects on further research emphasizing the importance of the support of public opinion about this type of treatment.

## Introduction

The discovery of modern technology has made it possible to generate any cell type outside the body in a highly controlled artificial environment using human induced pluripotent stem cells (iPSCs). iPSCs are derived by reprogramming adult somatic cells back to the pluripotent state in vitro by inducing a forced expression of specific transcription factors [1-6]. In addition to certain similarities with embryonic stem cells, iPSCs also show considerable differences, especially in the ethical aspect [6-9]. Although there are many possibilities offered for the potential application of these cells, there are always certain risks such as tumorigenesis [10-20].

Similar research was conducted which showed that the public is familiar with the concept of stem cells and that the respondents have a certain level of knowledge [21-27], but certain differences in relation to gender were also observed [28-30].

The aim of this paper is to examine the iPSC-related knowledge of general population and population of healthcare workers in relation to gender. Considering the between-gender differences observed in previous studies conducted on medical students or recent graduates, as well as the fact that women are generally more health conscious than men, it is hypothesized that the level of knowledge in this study will be higher among female participants, regardless of whether they are formally educated in medical field or not.

## Materials and methods

The research was designed as a cross-sectional cohort study in which we examined the level of information, knowledge, and attitudes of respondents from the general population and the population of healthcare professionals in relation to gender. The research was conducted with a validated anonymous questionnaire to assess information, knowledge, and attitudes about iPSCs donation, storage, and application, via e-mail and directly (using printed materials) during the period from September 1, 2021, to January 1, 2022.

Procedure

Prior to providing any responses to the survey questions, participants were informed of the anonymity and voluntary participation as part of the informed consent presented at the beginning of the survey. Prior to accessing the survey, the participants were given written information on the goal and the procedure of the study. There were no incentives provided to the participants, neither before, nor after the survey. Anonymity was prioritized and no identifiable information was collected. The procedure followed was in accordance with the ethical standards of the national responsible committees on human experimentation, as well as with the Helsinki Declaration, as revised in 2004.

Participants

The study included a sample of participants over 18 years of age, having the ability to read and write in Serbian language. The survey was conducted on the territory of two Serbian cities - Belgrade and Kragujevac. The sample of healthcare professionals (medical specialists) was selected by using a simple random sampling method. The questionnaire was sent in an electronic form to participants' email addresses extracted from the institution's employee database. The sample of the general population was collected by snowball technique. In this case, the questionnaire was provided to the participants in a paper form. The participants in the general population sample had various non-medical educational and experience-related backgrounds. The independent variables were gender, age, religion, socioeconomic status, and level of education, while the dependent variables were level of information, level of knowledge, and attitude towards iPSCs. The study was approved by the ethics committees of Faculty of Medical Sciences, University of Kragujevac (decision number 01-3148; March 19th, 2019) and Clinical Center Kragujevac (decision number 01119-1218; March 21st, 2019), both in Kragujevac, Serbia.

The survey

The structured validated Questionnaire for Evaluation of Awareness, Knowledge, and Attitudes towards Donation, Storage, and Application of iPSCs [31] was divided into three different sections: 1) demographic data; 2) information on attitudes towards iPSCs (assessed through 11 multiple choice questions); and 3) information on the level of information and knowledge about iPSCs (assessed through 12 Yes-No statements).

Demographic data collected included gender, age, religion, socioeconomic status, and level of education. These factors were compared to the data related to attitudes towards iPSCs and the level of information and knowledge about iPSCs.

The questionnaire was developed in English and was then translated into Serbian. After collecting all the surveys, the data was stored as an electronic Excel dataset in a reliable cloud-based storage system and afterward converted to the electronic statistical software compatible dataset file format. Three of the authors (JMG, SRR, MRJ) had access to the dataset and performed the statistical analysis. No need for data de-identification was present, due to the absence of privacy-related issues regarding the information present in the dataset. After completion of the study, the initial Excel dataset, as well as the converted one which was used for statistical analysis, were stored in the same cloud-based system. All of the survey-related procedures were overseen by Faculty of Medical Sciences, University of Kragujevac in Kragujevac, Serbia.

Complete statistical data analysis

Complete statistical data analysis was performed using IBM SPSS Statistics for Windows, Version 26 (Released 2019; IBM Corp., Armonk, New York, United States). Attribute variables were presented in the form of frequencies and percentages of individual categories, and statistical significance was tested using the chi-square test. The distribution of continuous variables was tested by the Kolmogorov-Smirnov test. Those variables were presented in the form of median and interquartile range (IQR). The significance of difference between continuous variables was tested by the Mann-Whitney U test. Analysis was estimated at the level of statistical significance of p≤0.05.

## Results

The study surveyed a total of 1,047 respondents, of which 560 (53.5%) were women and 487 (46.5%) were men. The age range of respondents was 31-60 years. No significant between-gender differences were observed in either age, religion, socioeconomic status, or education level, in both the healthcare professional group and general population group.

Level of knowledge and attitudes of respondents in the healthcare profession in relation to gender

Overall 371 healthcare professionals were included in the analysis, of which 171 (46.1%) were women and 200 (53.9%) were men. The gender-based differences in iPSC-related knowledge among healthcare professionals are shown in Table 1. Compared to men, women reported more frequently having heard of iPSCs, more often believed that these cells can be used in diabetes therapy, that they would accept treatment with these cells, more likely believed that healing can occur with these cells, and significantly more often believed that treatment with these cells is ethically and morally justified.

**Table 1:** Information on healthcare workers in relation to gender * Chi-Square test

	Men	Women	p*
Have you heard about induced pluripotent stem cells so-called iPSCs?			
Yes	139 (69.5%)	167 (97.7%)	<0.001
No	61 (30.5%)	/	
I don’t remember	/	4 (2.3%)	
Do you think iPSCs can be used to treat diabetes?			
Yes	81 (40.5%)	132 (77.2%)	<0.001
No	5 (2.5%)	5 (2.9%)	
I don’t know	114 (57.0%)	34 (19.9%)	
Would you accept treatment using iPSCs?			
Yes	72 (36.0%)	117 (68.4%)	<0.001
No	32 (16.0%)	/	
I don’t know	96 (48.0%)	54 (31.6%)	
Do you think there are risks when using iPSCs for treatment?			
Yes	137 (68.5%)	73 (42.7%)	<0.001
No	/	20 (11.7%)	
I don’t know	63 (31.5%)	78 (45.6%)	
Do you believe in healing using iPSCs?			
Yes	106 (53.0%)	105 (61.4%)	0.018
No	20 (10.0%)	5 (2.9%)	
I don’t know	74 (37.0%)	61 (35.7%)	
Is the treatment of IPSCs justified from the ethical and moral aspects?			
Yes	148 (74.0%)	152 (88.9%)	<0.001
No	10 (5.0%)	/	
I don’t know	42 (21.0%)	19 (11.1%)	
Would you leave your iPSCs in the state cell bank in Serbia or abroad?			
Serbia	104 (52.0%)	55 (32.2%)	<0.001
Abroad	39 (19.5%)	19 (11.1%)	
I don’t know	57 (28.5%)	97 (56.7%)	
Would you donate iPSCs for treatment purposes?			
Yes	132 (66.0%)	129 (75.4%)	0.061
No	/	/	
I don’t know	68 (34.0%)	42 (24.6%)	
Do you support research with iPSCs?			
I absolutely support	105 (52.5%)	98 (57.3%)	0.137
I support	91 (45.5%)	73 (42.7%)	
I don’t support	/	/	
I don’t support at all	/	/	
I don’t know	4 (2.0%)	/	
Do you agree with the research regarding the cloning of human tissues and organs?			
I absolutely agree	47 (23.5%)	36 (21.1%)	<0.001
I agree	95 (47.5%)	80 (46.8%)	
I don’t agree	13 (6.5%)	40 (23.4%)	
I don’t agree at all	28 (14.0%)	/	
I don’t know	17 (8.5%)	15 (8.8%)	
Are you interested in learning more about iPSCs?			
Yes	155 (77.5%)	165 (96.5%)	<0.001
No	8 (4.0%)	/	
Maybe	37 (18.5%)	6 (3.5%)	

Men more often believed that there are risks when using these cells. Also, when asked whether they would leave iPSCs for safekeeping in Serbia vs. abroad, the majority of men answered more often that they would leave them in Serbia, while women were mostly indecisive. However, the rest of the female participants more often decided that they would leave iPSCs for safekeeping in Serbia, similar to the answers provided by men.

Women responded statistically insignificantly more often that they would donate these cells and participants of both genders mostly supported research on these cells, with no significant between-gender differences. Women were significantly more likely to disagree with research related to the cloning of human tissues and organs than men. An interesting finding was that women were significantly more interested in finding out additional information about these cells compared to men (Table 1).

A statistically significant difference was observed in the distribution of correct answers to most statements in the questionnaire assessing iPSC-related knowledge between male and female healthcare workers (Tables 2, 3). The rate of correct answers among female healthcare workers ranged from 10.5% to 77.2% (Table 2), while the rate of correct answers among men ranged from 23.5% to 72.0%. The fewest correct answers among both women and men were obtained for the statement "Characteristics of pluripotent stem cells are self-renewal, potency, and differentiation” (Table 3).

**Table 2:** Correct answers to the questions in the knowledge part of healthcare workers in relation to gender * Chi-Square test

	Men	Women	p*
Induced pluripotent stem cells (iPSCs) are pluripotent stem cells derived by reprogramming the genome of terminally differentiated somatic cells.	86 (43.0%)	104 (60.8%)	0.001
IPSCs can be derived from different types of somatic cells.	117 (58.5%)	132 (77.2%)	<0.001
IPSCs can be differentiated only into bone, cartilage, and fatty tissue cells.	103 (51.5%)	120 (70.2%)	0.001
IPSCs can be isolated from the inner mass of a blastocyst.	80 (40.0%)	101 (59.1%)	<0.001
Characteristics of pluripotent stem cells are self-renewal, potency, and differentiation.	47 (23.5%)	18 (10.5%)	0.001
Somatic cell reprogramming is performed using gene vectors.	144 (72.0%)	98 (57.3%)	0.003
For reprogramming somatic cells, we used only the nonintegrative method with minicircular RNA.	50 (25.0%)	77 (45.0%)	<0.001
Transcription factors so called Yamanaka factors are Oct3/4, Sox2, FGF4, Klf.	69 (34.5%)	28 (16.4%)	<0.001
The transcription factors included in Thomson's modification are Oct4, Sox2, Nanog, Lin28.	83 (41.5%)	80 (46.8%)	0.359
You should donate or store your iPSCs in specialized institutions because they could be used for personal and scientific purposes.	106 (53.0%)	80 (46.8%)	0.233
iPSCs cannot be used in potential therapies for cardiovascular diseases, Parkinson's disease, diabetes, cancer, and leukemia because they would lead to tissue rejection.	110 (55.0%)	98 (57.3%)	0.732
There are risks of tumorigenesis in iPSC implantation.	89 (44.5%)	84 (49.1%)	0.432

**Table 3:** Distribution according to the number of correct answers to questions in the knowledge section in relation to the gender of healthcare workers * Chi-Square test

Number of correct answers	Men	Women	p*
0	4 (2.0%)	/	<0.001
1	/	2 (1.2%)	
2	22 (11.0%)	20 (11.7%)	
3	17 (8.5%)	29 (17.0%)	
4	26 (13.0%)	15 (8.8%)	
5	36 (18.0%)	14 (8.2%)	
6	18 (9.0%)	18 (10.5%)	
7	45 (22.5%)	22 (12.9%)	
8	19 (9.5%)	/	
9	/	11 (6.4%)	
10	13 (6.5%)	40 (23.4%)	
11	/	/	
12	/	/	

Also, the median number of correct answers per respondent from the group of female healthcare workers was 6 (IQR 3-9), while for men it was statistically insignificantly lower - 5 (IQR 4-7) (p=0.197). The range of correct answers per respondent was slightly different between genders. In the group of male healthcare workers, that number ranged from zero to ten correct answers, while in women the range was 1-10 correct answers. There were 23.4% of female and only 6.5% of male healthcare workers who gave 10 correct answers which is the highest number of correct answers given (Table 3).

Level of knowledge and attitudes of respondents of the general population in relation to gender

Overall 676 participants from the general population were included in the analysis, of which 389 (57.6%) were women and 287 (42.4%) were men. The awareness of those participants regarding iPSCs in relation to gender showed significant differences in almost all aspects assessed in the questionnaire. Women were significantly more likely to have heard of induced pluripotent stem cells compared to men. As many as 37.5% of men from the general population group have not heard of these cells. Almost equally, both genders believe that these cells can be used in diabetes therapy, but a significantly higher percentage of both genders do not know whether they can be used for this indication. Women would significantly more often accept treatment with these cells compared to men, but men more often believe that there are risks when using these cells. Women are more likely to believe that healing can occur with application of this kind of treatment and significantly more often believe that treatment with these cells is ethically and morally justified (Table 4).

**Table 4:** Information on the general population in relation to gender * Chi-Square test

	Men	Women	p*
Have you heard about induced pluripotent stem cells so-called iPSCs?			
Yes	142 (49.5%)	302 (77.6%)	<0.001
No	108 (37.6%)	65 (16.7%)	
I don’t remember	37 (12.9%)	22 (5.7%)	
Do you think iPSCs can be used to treat diabetes?			
Yes	111 (38.7%)	151 (38.8%)	0.877
No	7 (2.4%)	12 (3.1%)	
I don’t know	169 (58.9%)	226 (58.1%)	
Would you accept treatment using iPSCs?			
Yes	79 (27.5%)	260 (66.8%)	<0.001
No	20 (7.0%)	12 (3.1%)	
I don’t know	188 (65.5%)	117 (30.1%)	
Do you think there are risks when using iPSCs for treatment?			
Yes	166 (57.8%)	165 (42.4%)	<0.001
No	2 (0.7%)	18 (4.6%)	
I don’t know	119 (41.5%)	206 (53.0%)	
Do you believe in healing using iPSCs?			
Yes	134 (46.7%)	245 (63.0%)	<0.001
No	7 (2.4%)	5 (1.3%)	
I don’t know	146 (50.9%)	139 (35.7%)	
Is the treatment of IPSCs justified from the ethical and moral aspects?			
Yes	136 (47.4%)	252 (64.8%)	<0.001
No	2 (0.7%)	10 (2.6%)	
I don’t know	149 (51.9%)	127 (32.6%)	
Would you leave your iPSCs in the state cell bank in Serbia or abroad?			
Serbia	55 (19.2%)	173 (44.5%)	<0.001
Abroad	73 (25.4%)	128 (32.9%)	
I don’t know	159 (55.4%)	88 (22.6%)	
Would you donate iPSCs for treatment purposes?			
Yes	87 (30.3%)	282 (72.5%)	<0.001
No	18 (6.3%)	13 (3.3%)	
I don’t know	182 (63.4%)	94 (24.2%)	
Do you support research with iPSCs?			
I absolutely support	96 (33.4%)	176 (45.2%)	0.005
I support	134 (46.7%)	164 (42.2%)	
I don’t support	13 (4.5%)	8 (2.1%)	
I don’t support it at all	/	/	
I don’t know	44 (15.3%)	41 (10.5%)	
Do you agree with the research regarding the cloning of human tissues and organs?			
I absolutely agree	71 (24.7%)	63 (16.2%)	<0.001
I agree	87 (30.3%)	200 (51.4%)	
I don’t agree	77 (26.8%)	37 (9.5%)	
I don’t agree at all	/	8 (2.1%)	
I don’t know	52 (18.2%)	81 (20.8%)	
Are you interested in learning more about iPSCs?			
Yes	109 (38.0%)	263 (67.6%)	<0.001
No	53 (18.5%)	27 (6.9%)	
Maybe	125 (43.5%)	99 (25.4%)	

Compared to women, the majority of male responders were indecisive when it came to leaving the iPSCs for safekeeping in Serbia vs. abroad, and donation of iPSCs. Women would significantly more often donate these cells. Both genders most often supported research on these types of cells. Men were significantly more likely to disagree with research related to the cloning of human tissues and organs than women, while women were significantly more interested in learning more about these cells compared to men (Table 4).

Within the section assessing the level of information and knowledge about iPSCs, a statistically significant difference between men and women from the general population group was observed in the distribution of correct answers to three of the 12 statements (Table 5). Women significantly more often gave a correct answer to the statements "iPSCs can be derived from different types of somatic cells", "Transcription factors so-called Yamanaka factors are Oct3/4, Sox2, FGF4, Klf", while the correct answer to the statement "There are risks of tumorigenesis in iPSCs implantation" was significantly more often given by men (Table 5).

**Table 5:** Correct answers to questions in the knowledge part of the general population in relation to gender * Chi-Square test

	Men	Women	p*
Induced pluripotent stem cells (iPSCs) are pluripotent stem cells derived by reprogramming the genome of terminally differentiated somatic cells.	119 (41.5%)	186 (47.8%)	0.118
IPSCs can be derived from different types of somatic cells.	118 (41.1%)	203 (52.2%)	0.006
IPSCs can be differentiated only into bone, cartilage, and fatty tissue cells.	150 (52.3%)	184 (47.3%)	0.231
IPSCs can be isolated from the inner mass of a blastocyst.	86 (30.0%)	138 (35.5%)	0.155
Characteristics of pluripotent stem cells are self-renewal, potency, and differentiation.	80 (27.9%)	123 (31.6%)	0.335
Somatic cell reprogramming is performed using gene vectors.	141 (49.1%)	196 (50.4%)	0.806
For reprogramming somatic cells, we used only the nonintegrative method with minicircular RNA.	99 (34.5%)	136 (35.0%)	0.965
Transcription factors so-called Yamanaka factors are Oct3/4, Sox2, FGF4, and Klf.	46 (16.0%)	122 (31.4%)	<0.001
The transcription factors included in Thomson's modification are Oct4, Sox2, Nanog, and Lin28.	49 (17.1%)	62 (15.9%)	0.773
You should donate or store your iPSCs in specialized institutions because they could be used for personal and scientific purposes.	106 (36.9%)	117 (30.1%)	0.073
iPSCs cannot be used in potential therapies for cardiovascular diseases, Parkinson's disease, diabetes, cancer, and leukemia because they would lead to tissue rejection.	115 (40.1%)	149 (38.3%)	0.700
There are risks of tumorigenesis in iPSC implantation.	141 (49.1%)	132 (33.9%)	<0.001

The rate of correct answers among women from the general population group ranged from 15.9% to 52.2%, while the rate of correct answers among men ranged from 16.0% to 52.3%. The fewest correct answers among participants of both genders were obtained on the statements concerning the transcription factors related to iPCSs. The rate of correct answers higher than 50% in male participants was observed only to the statement "IPSCs can be differentiated only in bone, cartilage, and fatty tissue cells", while in women to statements "IPSCs can be derived from different types of somatic cells" and "Somatic cell reprogramming is performed using gene vectors" (Table 5).

Also, the median number of correct answers per respondent from the group of women was 4 (IQR 2-7) and similar in the group of males - 4 (IQR 1-7) (p=0.752). Regarding the minimum and maximum number of correct answers per respondent, in the general population male group, it ranged from zero to 11, while in women, the observed range was 0-12. Ten or more correct answers were observed in 5.9% of male participants from the general population, while in 12.4% of women (Table 6).

**Table 6:** Distribution according to the number of correct answers to questions in the knowledge section in relation to the gender of respondents from the general population * Chi-Square test

Number of correct answers	Men	Women	p*
0	54 (18.8%)	56 (14.4%)	<0.001
1	20 (7.0%)	39 (10.0%)	
2	13 (4.5%)	43 (11.1%)	
3	12 (4.2%)	44 (11.3%)	
4	46 (16.0%)	24 (6.2%)	
5	42 (14.6%)	36 (9.3%)	
6	23 (8.0%)	30 (7.7%)	
7	29 (10.1%)	22 (5.7%)	
8	20 (7.0%)	36 (9.3%)	
9	11 (3.8%)	11 (2.8%)	
10	13 (4.5%)	36 (9.3%)	
11	4 (1.4%)	10 (2.6%)	
12	/	2 (0.5%)	

Comparison of knowledge and attitudes between the general population group and healthcare workers, related to gender

Men's awareness of iPSCs in relation to occupation showed significant differences (Tables 1, 4). Male healthcare workers were significantly more likely to have heard of induced pluripotent stem cells compared to males from the general population (69.5% vs. 49.5%; p<0.001). Male participants in the healthcare worker group and those from the general population most often did not know that these cells can be used in diabetes therapy (57.0% vs. 58.9%), while approximately 40% of them in both groups believed that these cells can be used to treat diabetes (p=0.917). Male healthcare workers would significantly more often accept treatment with these cells compared to men from the general population (36.0% vs. 27.5%; p<0.001). Male healthcare workers more often believed that there are risks when using these cells, as many as 68.5% of them, while 57.8% of men from the general population gave the same answer (p=0.003). Male healthcare workers more often believed that there could be a cure found in this kind of therapy, compared to men from the general population group (53.0% vs. 46.7%; p<0.001). Male healthcare workers significantly more often believed that treatment with these cells is ethically and morally justified (74.0%), while men from the general population had the same view in only 47.4% of cases (p<0.001). Also, male healthcare workers answered more often that they would leave induced pluripotent stem cells for safekeeping in Serbia, compared to leaving them abroad (52.0% vs. 19.2%), while men from the general population would more often keep them (25.4%) when compared to male healthcare workers (19.5%) (p<0.001). Male healthcare workers were more likely to donate these cells compared to males from the general population group (66.0% vs. 30.3%; p<0.001). Male healthcare workers were more likely to support research with these cells compared to males from the general population group (52.5% vs. 33.4%; p<0.001). Male healthcare workers significantly more often agreed with research related to the cloning of human tissues and organs compared to males of the general population group (71.0% vs. 55.0%; p<0.001) and were significantly more interested in learning more about these cells compared to men in the general population group (77.5% vs. 38.0%; p<0.001).

Women's information regarding induced pluripotent stem cells in relation to occupation showed that there are also significant differences (Tables 1 and 4). Female healthcare workers were significantly more likely to have heard of induced pluripotent stem cells compared to females from the general population group (97.7% vs. 77.6%; p<0.001) and significantly more often believed that these cells can be used in diabetes therapy (77.2% vs. 38.8%; p<0.001). Female healthcare workers and those from the general population group would most often accept treatment with these cells (68.4% vs. 66.8%; p=0.067). Female healthcare workers, as often as those from the general population group, believed that there are risks when using these cells (42.7% vs. 42.4%; p=0.007). Women from both groups most often believed that healing can occur with application of these cells (61.4% vs. 63.0%; p=0.400). Female healthcare workers significantly more often believed that treatment with these cells was ethically and morally justified (88.9%), while women from the general population group had the same view in 64.8% of cases (p<0.001). Female healthcare workers would less often leave iPSCs for safekeeping in Serbia compared to the women from the general population group (32.2% vs. 44.5%; p<0.001). Female healthcare workers would donate these cells as often as women from the general population group (75.4% vs. 72.5%; p=0.053). Also, female healthcare workers were more likely to support research with these cells compared to females from the general population group (100.0% vs. 87.4%; p<0.001). Women from both groups mostly agree with research related to cloning of human tissues and organs (67.9% vs. 67.6%; p<0.001). Female healthcare workers were significantly more interested in learning additional information about these cells compared to women in the general population group (96.5% vs. 67.6%; p<0.001).

A significant difference was also observed between male healthcare workers and those from the general population group, in terms of the distribution of correct answers to most questions assessing the level of knowledge about iPSCs (Tables 2, 3, 5, and 6). The rate of correct answers among male healthcare workers ranged from 23.5% to 72.0%, while the rate of correct answers among males of the general population group was from 16.0% to 52.3%. The respective p values for comparison of the number of correct answers to the 12 knowledge-related questions between male healthcare professionals and males from the general population group were: 0.807, <0.001, 0.941, 0.028, 0.329, <0.001, 0.033, <0.001, <0.001, <0.001, 0.002, and 0.361. Male healthcare workers more often gave correct answers to questions 2, 4, 6, 8, 9, 10, and 11, while men from the general population more often correctly answered only to knowledge question 7.

When comparing female healthcare workers and those from the general population group, a statistically significant difference in the distribution of correct answers to most questions was also observed (Tables 2, 3, 5, and 6). The rate of correct answers among female healthcare workers ranged from 10.5% to 77.2%, while the range of correct answers proportion among women in the general population was 15.9-52.2%. The respective p values for comparison of the proportion of correct answers to the 12 knowledge-related questions between female healthcare professionals and females from the general population group were: 0.006, <0.001, <0.001, <0.001, <0.001, 0.156, 0.030, <0.001, <0.001, <0.001, <0.001, and 0.001. Female healthcare workers more often gave correct answers to questions 1, 2, 3, 4, 7, 9, 10, and 11, while women from the general population more often correctly answered knowledge-related questions 5 and 8.

Also, the average number of correct answers per respondent from the group of male healthcare workers was significantly higher compared to men from the general population group (Med 5 vs. 4; p<0.001), and the average number of correct answers per respondent from the group of female healthcare workers was significantly higher compared to women from the general population group (Med 6 vs. 4; p<0.001)

## Discussion

Breakthroughs in stem cell research (SCR) and regenerative medicine (RM) have attracted significant public attention around the world. At the same time, the information, knowledge, and attitudes of the public can have multiple effects on further research in the sense of continuation or interruption. Therefore, the support of public opinion should not be neglected.

Studies similar to the current one were previously conducted in China, where the knowledge and attitudes of medical workers about stem cells were examined. Just over half of the participants were familiar with iPSCs, and 78.9% of the participants expressed high levels of interest in stem cells, especially those aged between 25 and 30 years who were well-educated. In addition, 54.2% supported the research of iPSCs upon explanation of nature, which also correlated with participants’ age, education level, and geographic region, but not with gender [21,22].

In Japan, public opinion about iPSCs has been surveyed for several years by Shineha et al. The study results showed that the general and scientific population in Japan is well-informed about iPSCs and supports further research on these cells. Ishihara and associates conducted a study with students and found a significant difference between females and males. Men showed better understanding of iPSCs and RM, greater interest, and expressed greater support for further research. On the other hand, more women showed interest and concern but were ready for additional education regarding these stem cells [23-27].

Shineha et al. conducted a pilot study, an international comparison of public attitudes toward SCR and RM, with an internet questionnaire among people in six countries: Japan, South Korea, the United States, the UK, Germany, and France. Their findings showed the diversity of interests in RM among the six countries, in terms of user pragmatism, governance and handling of RM, risk, benefit, and scientific interests. The trust of experts in SCR and RM varies in each country and may be influenced by the political, social, cultural, and media contexts of SCR and RM in each country [27].

Between-gender differences have rarely been observed in recent publications, almost exclusively in medical sciences students or medical professionals.

Two studies published in 2019 and 2021 assessed knowledge and attitudes towards stem cells and the significance of their medical application, but among healthcare sciences students (no between-gender difference observed) [29] and recent graduates of dental schools (higher knowledge score in females) [28].

Also, Abdulrazeq et al. conducted an interactive teaching intervention course on medical students’ knowledge and attitudes toward stem cells, their therapeutic uses, and potential research applications. They also examined the gender differences and found that men's total knowledge score was higher than women's. However, after the intervention, differences in stem cell knowledge between men and women decreased after the intervention course, and gender differences were not statistically significant [30].

Our study represents the first comparison of knowledge about and attitudes toward iPSCs between men and women in the general population. This study showed that the public is familiar with stem cells, but that there are significant differences in knowledge about iPSCs between the genders, both between members of the general population and the population of healthcare workers. Women from both populations were better informed, would more often agree to treatment with these cells, and significantly more often considered that therapy with these cells is ethical and morally justified. They also more often supported further research and were ready to learn more about these cells compared to men. When we compared men and women from the healthcare professional group and general population group, male and female healthcare workers were better informed, and showed greater knowledge. Male healthcare workers were significantly more likely to support research with iPSCs compared to men from the general population group, while women showed no significant differences between the groups. Female healthcare workers were significantly more interested in additional education about these cells.

In our study, a significantly higher percentage of women were willing to donate iPSCs, compared to men. This correlates to previously published findings [32,33]. Additionally, in a 2016 study investigating stem cell donation behaviors among medical students, positive predictors for participation in the bone marrow registry included prior blood donation and female gender [34]. This can be related to organ transplantation. However, no significant differences are observed between females and males in their willingness to donate their organs after death [35], although males are more commonly selected by transplant centers but are underrepresented on worldwide registries, because of better survival, reduced risk of chronic graft-versus-host disease and higher cell count yields with peripheral blood stem cell collections [33].

The observational and cross-sectional design of this study limits the implications of its results only in the present time. Also, it only investigates the association between gender and attitudes and information/knowledge levels of participants, with limited ability to report specific causalities. The snowball method used for the sampling of participants in the general population group represents another shortcoming by reducing the representativeness of the sample. Our study did not analyze other factors which information was collected by this questionnaire, including age, religion, socioeconomic status, and level of education, although that fact does not significantly influence the power of the results observed in our study, due to the matching adjustments and the absence of statistically significant differences among genders in terms of the remaining demographic variables collected. Finally, the sampling was conducted in two provinces in Serbia (both university centers) which limits the application of the obtained results only to similar environments in Serbia. These aspects should be improved in future research, with the development of more detailed questionnaires that will include more demographic and other data potentially influencing participants' perception towards iPSCs.

## Conclusions

Women are better informed about iPSCs and have a more positive attitude towards that kind of treatment, regardless of the fact whether they are healthcare professionals or not, which confirms the initial hypothesis of the study. As expected, healthcare professionals are better informed about iPSCs, compared to general population. These results are very useful and can contribute to future research on stem cells, motivate the scientific population in the direction of further research on induced pluripotent stem cells, and encourage the public to have additional education about these cells.
